# It is theoretically possible to avoid misfolding into non-covalent lasso entanglements using small molecule drugs

**DOI:** 10.1371/journal.pcbi.1011901

**Published:** 2024-03-12

**Authors:** Yang Jiang, Charlotte M. Deane, Garrett M. Morris, Edward P. O’Brien

**Affiliations:** 1 Department of Chemistry, Pennsylvania State University, University Park, Pennsylvania, United States of America; 2 Oxford Protein Informatics Group, Department of Statistics, University of Oxford, 24-29 St Giles’ Oxford, OX1 3LB United Kingdom; 3 Bioinformatics and Genomics Graduate Program, The Huck Institutes of the Life Sciences, Pennsylvania State University, University Park, Pennsylvania, United States of America; 4 Institute for Computational and Data Sciences, Pennsylvania State University, University Park, Pennsylvania, United States of America; Korea Institute for Advanced Study, REPUBLIC OF KOREA

## Abstract

A novel class of protein misfolding characterized by either the formation of non-native noncovalent lasso entanglements in the misfolded structure or loss of native entanglements has been predicted to exist and found circumstantial support through biochemical assays and limited-proteolysis mass spectrometry data. Here, we examine whether it is possible to design small molecule compounds that can bind to specific folding intermediates and thereby avoid these misfolded states in computer simulations under idealized conditions (perfect drug-binding specificity, zero promiscuity, and a smooth energy landscape). Studying two proteins, type III chloramphenicol acetyltransferase (CAT-III) and D-alanyl-D-alanine ligase B (DDLB), that were previously suggested to form soluble misfolded states through a mechanism involving a failure-to-form of native entanglements, we explore two different drug design strategies using coarse-grained structure-based models. The first strategy, in which the native entanglement is stabilized by drug binding, failed to decrease misfolding because it formed an alternative entanglement at a nearby region. The second strategy, in which a small molecule was designed to bind to a non-native tertiary structure and thereby destabilize the native entanglement, succeeded in decreasing misfolding and increasing the native state population. This strategy worked because destabilizing the entanglement loop provided more time for the threading segment to position itself correctly to be wrapped by the loop to form the native entanglement. Further, we computationally identified several FDA-approved drugs with the potential to bind these intermediate states and rescue misfolding in these proteins. This study suggests it is possible for small molecule drugs to prevent protein misfolding of this type.

## Author summary

A variety of diseases are caused by protein misfolding. Therefore, the recent evidence suggesting there is an entire unexplored class of protein misfolding that may be widespread opens the possibility they contribute to disease and may be therapeutic targets. Here, we bypass the question of what diseases such misfolding may contribute to and ask whether it is even possible to restore proper folding and function to proteins that misfold in this manner. We tried the most obvious strategy first: in the computer simulations we created a drug that binds to the folded, native entanglement. The rationale being that this would thermodynamically stabilize the native state relative to misfolded states, and thereby shift the population of molecules to the folded states. But thermodynamic reasoning neglects the folding pathways and kinetics connecting metastable states on the energy landscape, and this strategy had the unintended consequence of slowing down native state formation by stabilizing the entanglement loop without the threading segment piercing it, resulting in the loss of the native entanglement and formation of a non-native entanglement. Building on this observation, we took a different route, and designed a drug that would delay formation of the entanglement loop allowing more time for the threading segment to position itself and allow proper folding of the native entanglement. While this study was carried out using coarse-grained structure-based models, the results indicate it is possible in principle for drugs to be designed to avoid such misfolding and suggest our second design strategy is more likely to work in future experiments.

## Introduction

**Fig 1:**
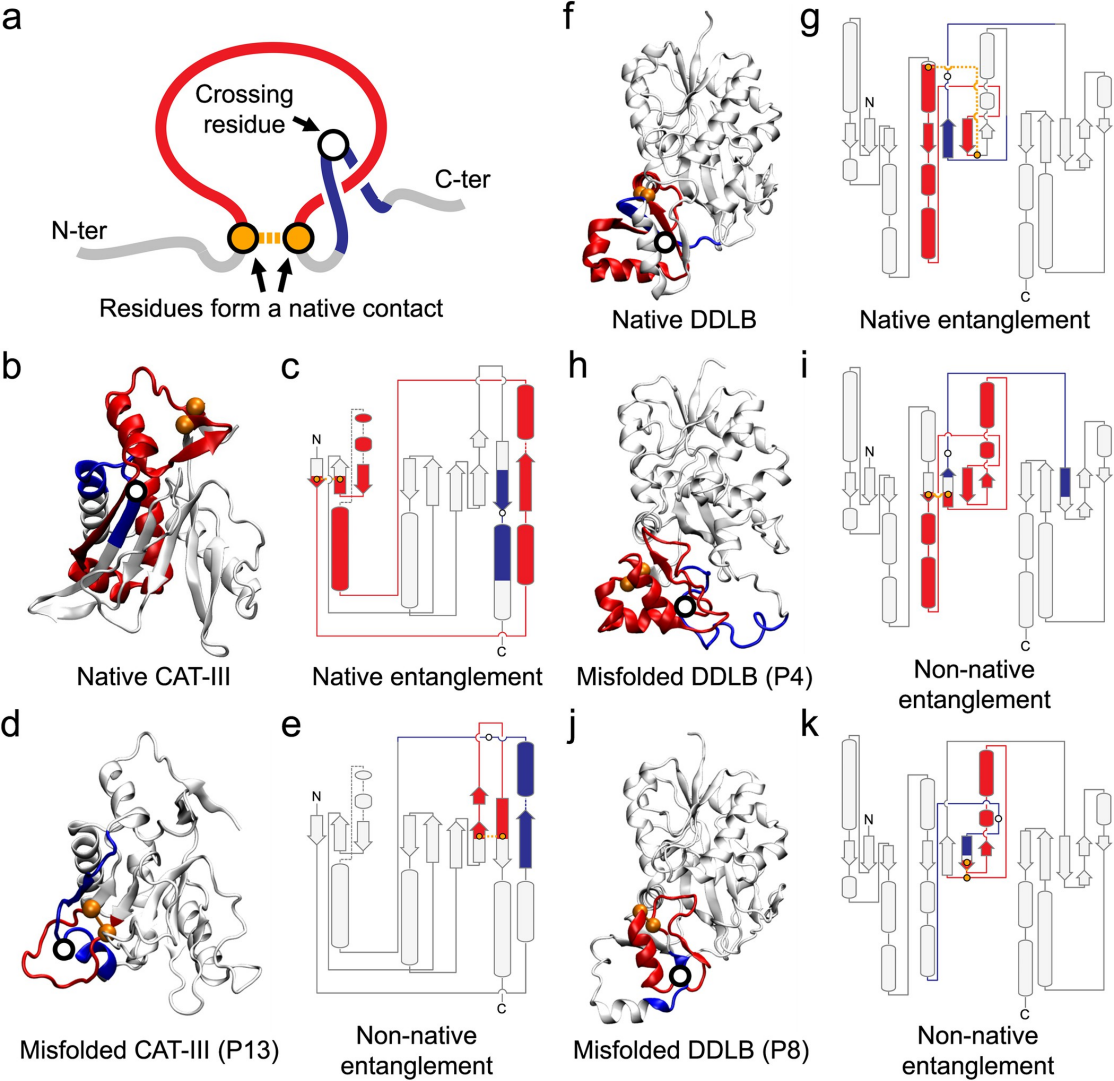
Non-covalent lasso entanglements in native and misfolded proteins. (a) Topology diagram illustrating the non-covalent lasso, where a closed loop (red) formed by a native contact (orange) is threaded through by a segment (blue), with the crossing residue depicted as a white circle. The remaining regions of the protein are depicted in gray. (b) Native structure of CAT-III monomer (PDB 3CLA) highlighting the native entanglement (loop: 5–78; crossing: 190). (c) Topology diagram representing the native entanglement in CAT-III. (d) Misfolded structure of CAT-III state P13, obtained from the previous work [3], with the non-native entanglement highlighted (loop: 169–184; crossing: 35). (e) Topology diagram representing the non-native entanglement in CAT-III state P13. (f) Native structure of DDLB (PDB 4C5C) highlighting the native entanglement (loop: 98–146; crossing: 179). (g) Topology diagram representing the native entanglement in DDLB. (h) Misfolded structure of DDLB state P4, obtained from the previous work [3], with the non-native entanglement highlighted (loop: 117–177; crossing: 186). (i) Topology diagram representing the non-native entanglement in DDLB state P4. (j) Misfolded structure of DDLB state P8, obtained from the previous work [3], with the non-native entanglement highlighted (loop: 145–174; crossing: 141). (k) Topology diagram representing the non-native entanglement in DDLB state P8. The layout of secondary structure elements in the topology diagrams was obtained from PDBe server [8].

A new class of protein misfolding has recently been predicted to exist based on coarse-grained and all-atom models of protein folding. This misfolding involves the formation of either non-native intramolecular entanglement in which backbone segments intertwine with one another, or the failure to form intramolecular entanglements that are present in the folded state. Specifically, non-covalent lassos, which are geometrically defined by the formation of a loop closed by a native contact and is threaded through by another segment of the protein chain (Fig 1A), have been observed in all-atom and coarse-grained protein folding simulations. Computational investigations suggest that this type of misfolding can occur in half of all globular protein in *E*. *coli* [1], can bypass proteostasis quality control machinery[1,2], and can cause long-term alterations in enzymatic activity upon introduction of synonymous mutations [3]. Several biochemical and mass-spectrometric comparisons are consistent with the existence of these soluble, misfolded states [1–3]. Further, cryo-EM structures recently revealed RNA molecules can misfold into non-covalent lasso entanglements [4,5] and coarse-grained RNA simulation models can recapitulate such misfolding [6].

The possibility of a new class of monomeric protein misfolding is exciting because it offers the possibility of new therapeutic drug targets. Indeed, in a recent study, evidence was provided that these misfolded states cause a loss-of-function by reducing enzymatic activity [1,3]. In this study we address a very narrow and specific question. If changes of entanglement can cause protein misfolding, is there any theoretical way to avoid such misfolding using small molecule drugs under idealized conditions? We define ideality as a small molecule compound that has perfect specificity, zero promiscuity, and only binds one specific location in the protein. If we cannot avoid misfolding under these ideal conditions, then misfolding is unlikely to be avoided under more complex, realistic conditions. Such ideality therefore represents a necessary but not sufficient condition for demonstrating it is possible to design small molecule compounds that can avoid such misfolding. This study represents a first step towards a longer-term goal of designing drug therapies that target change-of-entanglement misfolding.

It is possible to achieve such ideality using structure-based (Gō) force-fields, in which the drug molecule only has attractive interactions for the target binding-site residues and repulsive interactions otherwise. Further, since we aim to simulate the entire unrestrained folding process, and all-atom transferable force fields are unable to do this for proteins of more than 200 residues [7], we must coarse-grain the structural representation of the protein. In this case each amino acid residue is represented as a single interaction site and the drug molecule as nine interaction sites. This allows us to map out how the folding reaction network changes in the presence of this ideal drug.

With this approach, we demonstrate it is theoretically possible to design drugs to avoid misfolding into these kinetic traps for two proteins, and we *in silico* screen several already FDA-approved drugs that we predict could bind to the target sites on these proteins. The drug design strategy we present can be applied to any protein of interest.

## Results

### Misfolding arises from loss of a native entanglement and formation of a non-native entanglement

**Fig 2:**
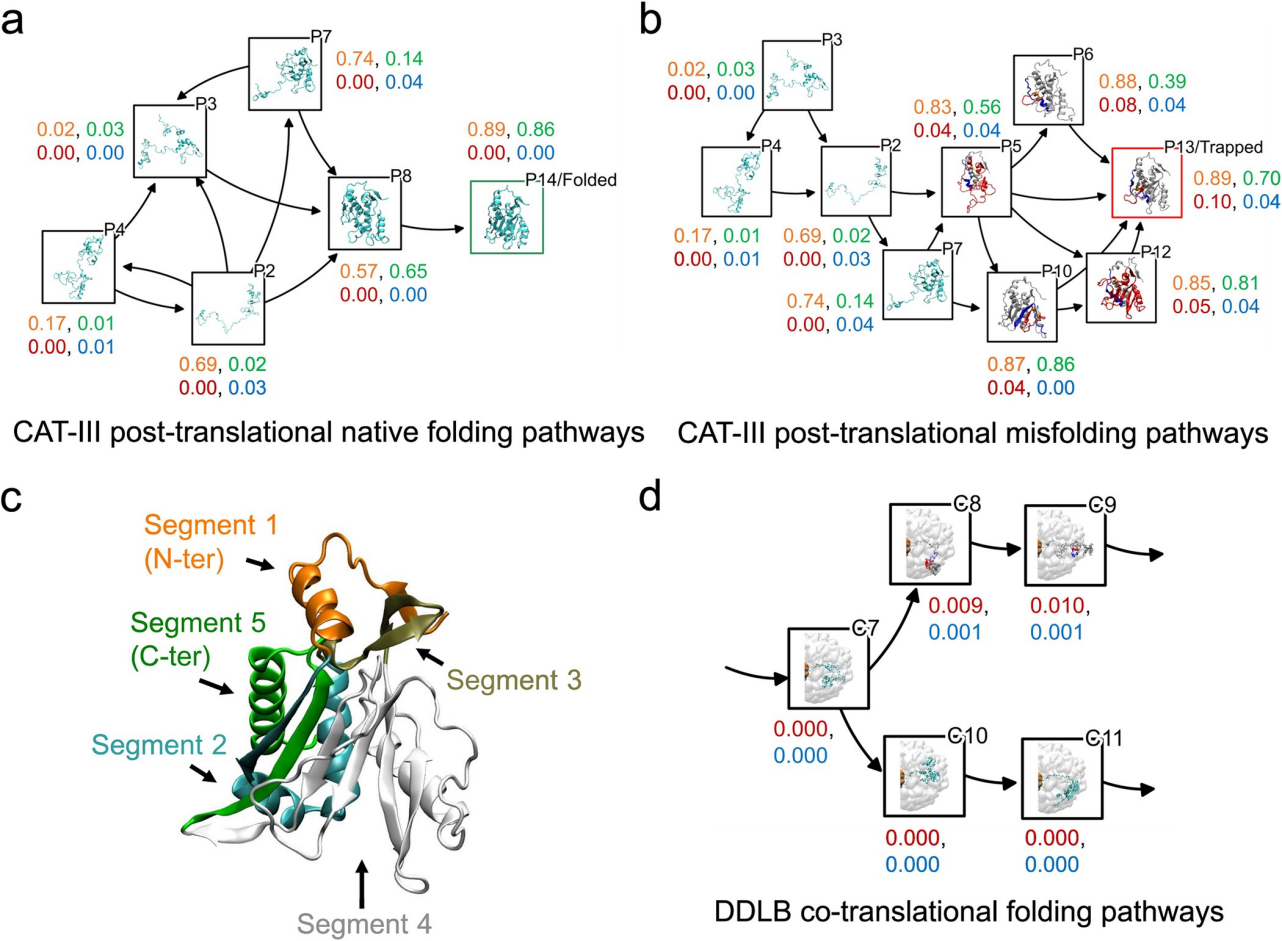
Non-native entanglement was formed due to loss of the native entanglement. The 80% most probable CAT-III post-translational pathways for (a) native folding and (b) misfolding are shown as a transition network with arrows indicating transitions from one state to another. Each node represents a state, and a representative structure of each state is presented, with the state ID at the top right corner. Structures without gain of non-native entanglements are shown in cyan, while the non-native entanglements are shown in red on the closed loop and blue on the threading segment, with the rest of the protein in white. Four values are presented near each node, representing 〈*Q*_1|3_〉 (orange), 〈*Q*_2|5_〉 (green), 〈*G*_gain_〉 (red) and 〈*G*_loss_〉 (blue). (c) Native structure of CAT-III (PDB ID: 3CLA) with five segments represented in different colors. (d) A portion of the co-translational folding pathways of DDLB starting from state C7, which is separated into a misfolding pathway (top) and a native folding pathway (bottom). All the representations are the same as in panels (a) and (b), except for the inclusion of a white surface to depict the ribosome in each state, as non-native entanglements form co-translationally for this protein [3].

To design a drug that avoids misfolding we need to understand the pathways leading to those states. The misfolded entangled states of CAT-III are all formed post-translationally in a complex folding network (see Fig 6A of Ref. [3]). We analyzed the post-translational folding trajectories from our previous work [3], which included CAT-III synthesized either quickly or slowly by the ribosome. We divided the folding pathways into two categories: those leading to the native state (labeled P14 in Fig 2A), which we refer to as native folding pathways, and those leading to the misfolded entangled state (P13, Fig 2B), referred to as misfolding pathways. In the native folding pathways, the protein first converted states P2, P3, and P7 to a near-native intermediate state P8 without forming any non-native entanglements. From this intermediate state, the protein then folded to the native state P14. Interestingly, more than 70% of the native folding pathways passed through states P3 and P8 before reaching the native state P14.

In contrast, the misfolding pathways were characterized by the conversion of states P2 and P7 to misfolded entangled states, such as P5, P6, P10, and P12, before eventually reaching the kinetically trapped state P13. Specifically, we found that 60% and 22% of the misfolding pathways passed through states P2 and P7, respectively, before the protein became entangled.

To understand the formation of native and non-native entanglements at an even higher resolution, we calculated the fraction of native contacts formed between the segments that make up the entanglements. We divided the primary structure of CAT-III into five segments, as shown in Fig 2C. The contacts between segments 1 and 3 are responsible for forming the closed loop of the native entanglement (as shown in Fig 1B and 1C) and correctly positioning the threading segment of the *non-native* entanglement (as shown in Fig 1D and 1E). On the other hand, the contacts between segments 2 and 5 help to correctly position the threading segment of the *native* entanglement and facilitate the formation of the closed loop of the non-native entanglement. We calculated the average values of the fraction of native contacts formed between segments 1 and 3 (denoted 〈*Q*_1|3_〉) and between segments 2 and 5 (denoted 〈*Q*_2|5_〉) across all structures within each metastable state shown in Fig 2. Additionally, we computed the average values of the fraction of gain of non-native entanglements (denoted 〈*G*_gain_〉, Eq 2) and the fraction of loss of native entanglements (denoted 〈*G*_loss_〉, Eq 3) to monitor changes of entanglement along the folding pathways.

We find that states P2 and P7 had high 〈*Q*_1|3_〉 values (0.69 and 0.74, respectively) and low 〈*Q*_2|5_〉 values (0.02 and 0.14, respectively). Both states also had a loss of the native entanglement demonstrated by 〈*G*_gain_〉 being zero and 〈*G*_loss_〉 being non-zero. These results indicate that in states P2 and P7, the closed loop of the native entanglement is already formed, but the threading segment is not yet in the correct position leading to a loss of the native entanglement. In the native folding pathways, states P2 and P7 tend to open the native closed loop and convert to state P3 as 〈*Q*_1|3_〉 and 〈*Q*_2|5_〉 are very close to zero. From State P3 the protein can transition to form the native entanglement and ultimately reach the native state P14. In contrast, in the misfolding pathways, states P2 and P7 tend to maintain the closed loop of the native entanglement and wrap the native threading segment around it, rather than allowing the threading segment to pierce through it, evidence by the transitions to entangled state P5 with non-zero 〈*G*_gain_〉 and 〈*G*_loss_〉 values. This failure to form the native entanglement leads to the formation of the non-native entanglement, where the threading segment in the native entanglement serves as the closed loop in the non-native entanglement.

In contrast to CAT-III, the formation of non-native entanglements in DDLB’s domain III (see Fig 1H–1K) occurred during protein synthesis and co-translational folding, where folding and misfolding pathways clearly diverged (see Fig 6B in Ref [3]). Nevertheless, as in CAT-III, these non-native entanglements resulted from the failure to form the native entanglement (see Fig 1F and 1G), which was demonstrated by the loss of native entanglement observed in co-translational misfolded intermediate states C8 and C9 with non-zero 〈*G*_loss_〉 values (see Fig 2D). Thus, the failure to form the native entanglement results in the formation of the non-native entanglement observed in most of the misfolded metastable states of both CAT-III and DDLB.

### A Failed Drug-design Approach: Stabilizing the native entanglement

**Fig 3:**
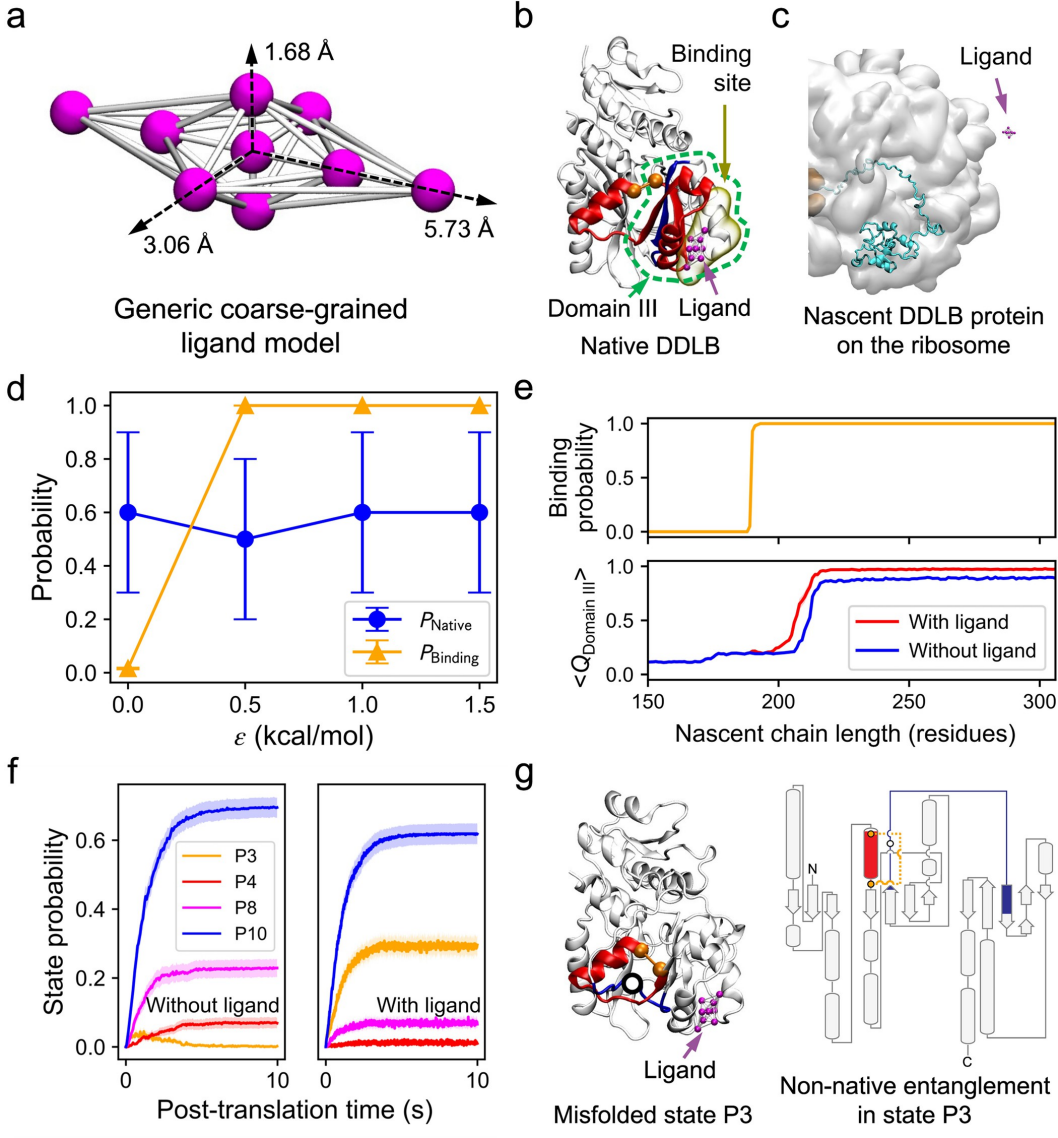
Simply stabilizing the native entanglement is not an effective way to rescue misfolded DDLB proteins. (a) The 3D structure of a coarse-grained ligand model. The interaction sites are colored magenta, and the covalent bonds are colored white. The principal axes of the mass distribution tensor are indicated by dashed arrows, with the maximum bond length from the central bead listed. (b) Native DDLB protein structure highlighting the native entanglement in Domain III (green dashed circle) and the predicted ligand binding site (yellow). The CG ligand (magenta) is shown bound to the target site. Entanglements are depicted as in Fig 1. (c) An initial structure of the nascent DDLB protein of 190 residues (cyan) on the ribosome (white) in the presence of the ligand (magenta). (d) Probabilities of natively entangled structure formation at length 230 (*P*_Native_, blue) and ligand binding (*P*_Binding_, orange) vs. the interaction energy *ε*_*ij*_ (see Method). Error bars represent the 95% confidence intervals (CIs) estimated by bootstrapping. (e) Ligand binding probability vs. nascent chain (NC) length (top) and the averaged fraction of native contact formed in Domain III vs. NC length with (red) and without (blue) ligand present (bottom). Transparent stripes represent 95% CIs estimated by bootstrapping. (f) Probabilities of forming misfolded entangled states P3 (orange), P4 (red), P8 (magenta), and native state P10 (blue) as a function of post-translational time for the slow DDLB variant with (right) and without (left) ligand present. Transparent stripes represent 95% CIs estimated by bootstrapping. (g) A structure of the enriched misfolded state P3 with the ligand bound (left) and the non-native entanglement topology diagram (right, loop: 98–116; crossing: 186). Entanglements are depicted as in Fig 1.

Given that the formation of the non-native entanglement results from the failure to form the native entanglement, we hypothesized a way to prevent protein misfolding is to promote the formation of the native entanglement. One way to achieve could be by stabilizing the native entanglement during protein synthesis and folding. To test this strategy we developed a generic coarse-grained (CG) model for small molecule ligands (Fig 3A) and examined its ability to promote DDLB folding, which has a well-defined ligand binding site in its natively entangled domain (Domain III) as identified by AutoSite [9] (Fig 3B). The binding pose of the CG ligand was designed to appropriately fit the predicted binding site. To set a realistic binding affinity of the CG ligand for the protein in the force field, we conducted simulations of DDLB on a translationally *arrested* ribosome at a nascent chain length of 230 residues (where Domain III can fold) in the presence of the ligand with varying non-bonded interaction strengths (see Method section *Binding affinity scan*). As expected, the probability of ligand binding increased monotonically as the interaction energy strength (Lennard-Jones well-depth) between the ligand and its binding site was increased. However, contradicting our prediction, the fraction of folded protein molecules was largely unchanged, staying around 0.6 (Fig 3D), indicating that binding of the ligand at the target site does not improve the yield of non-entangled nascent proteins on arrested ribosomes. Nevertheless, we chose a binding affinity of *ε*_*ij*_ = 1.0 kcal/mol for the subsequent simulations involving continuous protein synthesis in which co- and post-translational folding of DDLB can occur.

We simulated the co-translational (with 100 trajectories) and post-translational folding (with 1,000 trajectories) of the slow-translating mRNA variant in the presence of the ligand. We find the ligand successfully binds its target site on DDLB and stabilized its structure, as demonstrated by the 100% binding probability and the increased fraction of native contacts formed within Domain III, respectively (Fig 3E). However, as shown in Fig 3F, this did not lead to an increase in the native state (P10) population at the end of post-translational folding. Instead, it shifted the population to another entangled misfolded state P3, which is another type of non-native entanglement, as depicted in Fig 3G. Thus, targeting the native entanglement for stabilization is not a general solution to avoiding this type of misfolding.

### Most entanglements form by wrapping the loop around the threading segment

**Fig 4:**
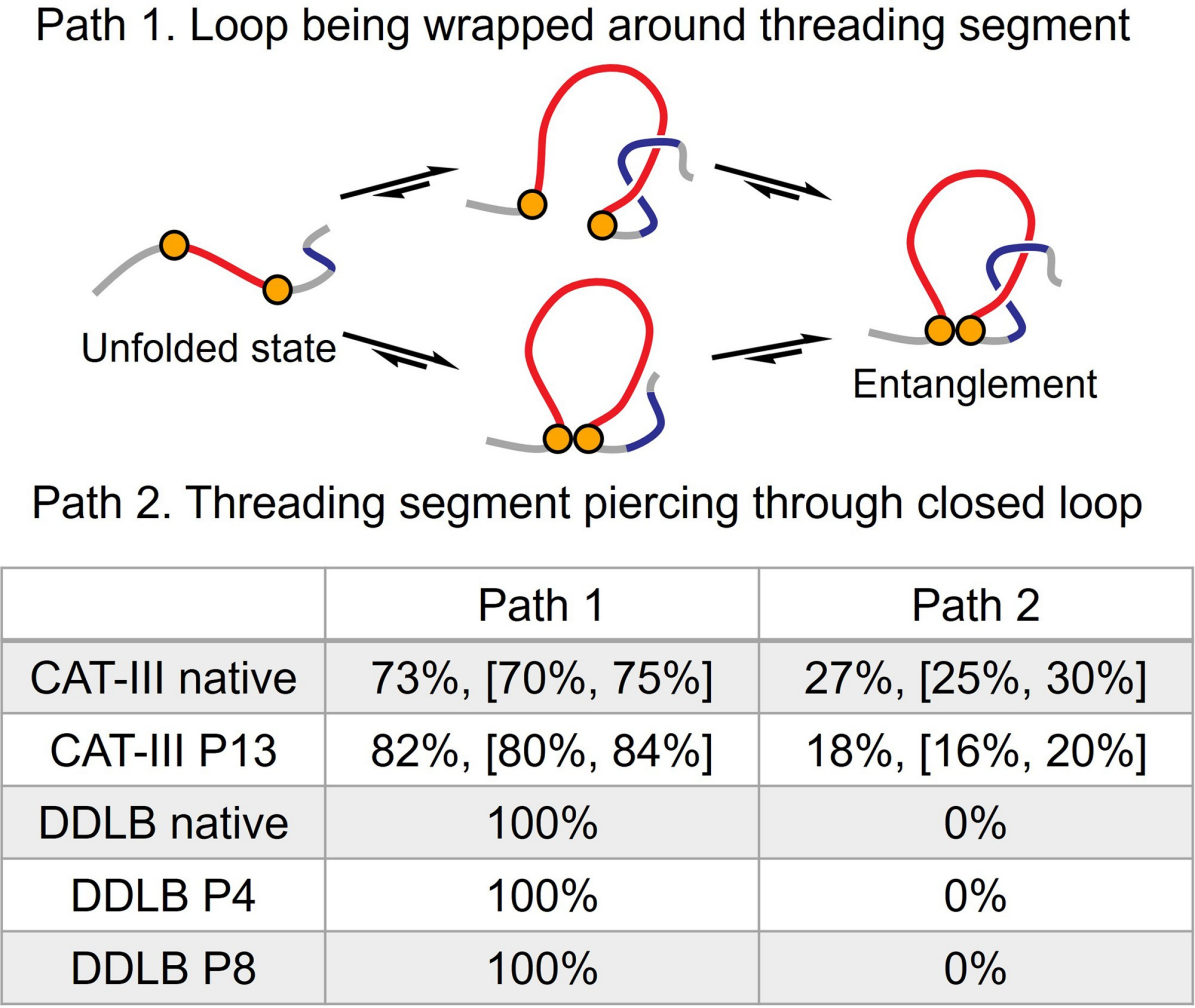
Two parallel paths to forming an entanglement involving a single threading event. Orange circles represent the two residues forming the native contact that closes the loop. The loop and threading segment are shown in red and blue, respectively. A table is presented at the bottom showing the probabilities of both paths utilized by the native and misfolding pathways, respectively. The 95% confidence intervals were estimated by bootstrapping and are presented in the bracket for the probability value less than 100%.

To find an alternative drug design strategy, we reexamined the entanglement formation pathways from Ref. [3]. In the folding and misfolding pathways, we find that the primary path of forming an entanglement involves first placing the threading segment in the correct position and then closing the loop by wrapping it around the threading segment (as shown in Fig 4, Path 1). This primary path occurs in 73% and 82% of trajectories that form native and non-native entanglements in CAT-III, respectively, and is utilized in all trajectories for DDLB. A secondary path was also observed in which the threading segment pierces the loop after the loop has already formed (as shown in Fig 4, Path 2). However, this secondary path occurs only in 27% and 18% of cases for the formation of the native and non-native entanglements, respectively, in CAT-III and does not occur in DDLB’s folding pathways.

### Alternative Drug-design Approach: Delaying formation of the native loops avoids misfolding

Since the primary pathway for forming the native entanglement involves wrapping the loop around the correctly placed threading segment, we hypothesized an alternative drug design strategy: delaying loop closure should allow more time for the threading segment to be synthesized and properly positioned for the loop to wrap around it, thereby promoting faster native structure formation. That is, this strategy aims to increase the flux through Path 1 in Fig 4. To test this hypothesis, we developed a CG ligand that targets intermediate structures of a protein segment in the native closed loop of DDLB (residues 126 to 174) and CAT-III (N-terminal residues 1 to 35), respectively. The ligand is designed to stabilize non-native tertiary structures involving the native loop segments, thereby preventing the formation of the native closed loop during the initial stages of protein folding.

To identify potential non-native tertiary structures of those DDLB and CAT-III segments we used structure prediction tools AlphaFold2[10,11], PEP-FOLD3[12] and QUARK [13,14]. The top 5 structures predicted by AlphaFold2 were structurally highly similar and were distinct from those predicted by the other tools, as demonstrated by pairwise root-mean-squared-distance (RMSD) values shown in S1A Fig for DDLB and S1D Fig for CAT-III. The AlphaFold2 structures resembled the native structure found in the PDB for both segments (S1B Fig for DDLB and S1E Fig for CAT-III), indicative of AlphaFold2’s training on native structures. For DDLB, the structures predicted by PEP-FOLD3 have larger deviations from the native structure than those predicted by QUARK and form non-native tertiary structures (S1B Fig). For CAT-III, the structures predicted by both PEP-FOLD3 and QUARK have large deviations from the native structure and contain non-native tertiary structures (S1E Fig). As our goal is to stabilize non-native structures, we discarded the native-like structures from AlphaFold2 and used AutoSite [9] to identify ligand binding sites in the non-native structures (S1C Fig for DDLB and S1F Fig for CAT-III).

**Fig 5:**
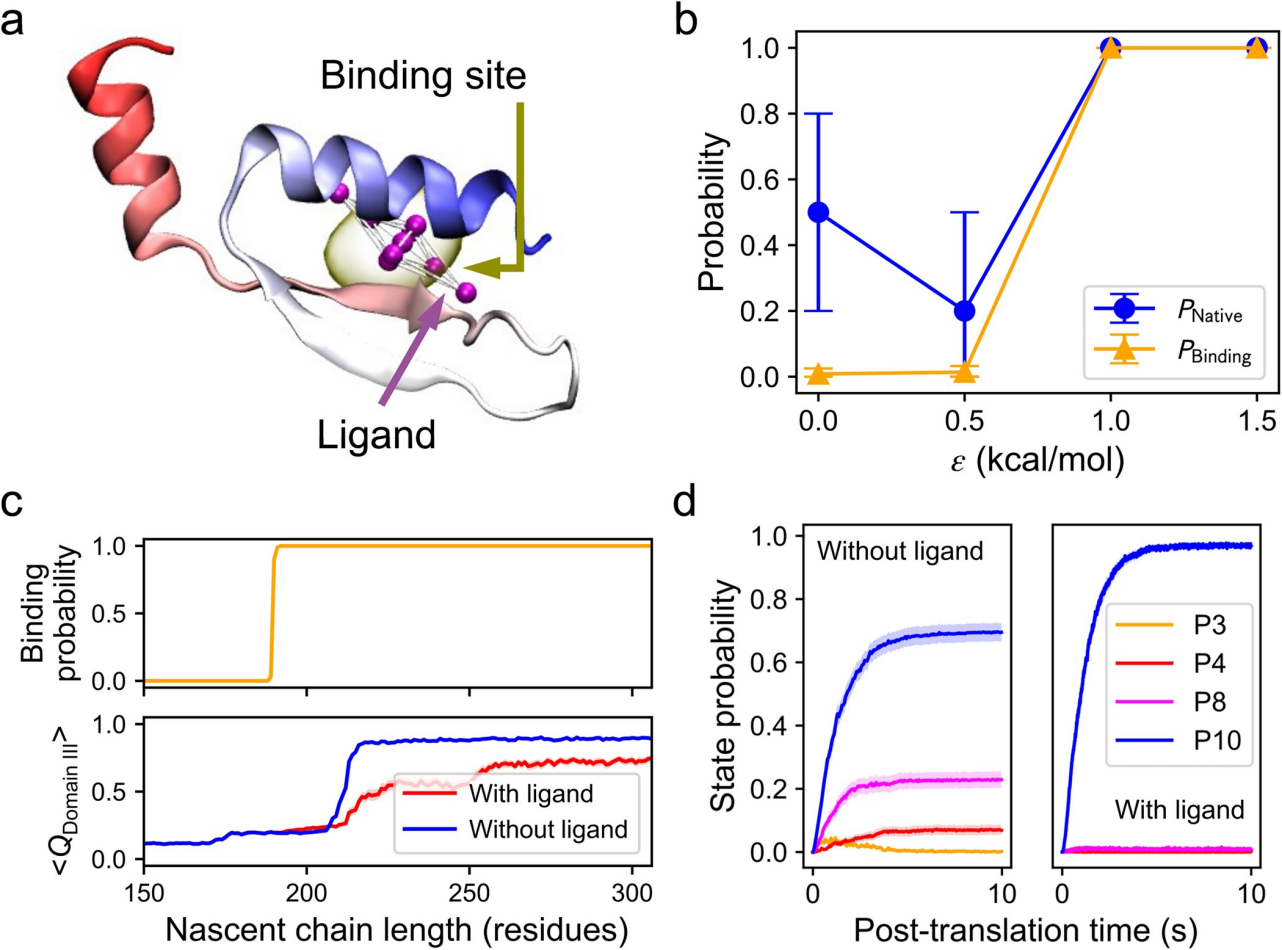
Ligand destabilizing native closed loop rescues misfolded DDLB. (a) Predicted non-native structure of DDLB segment 126 to 174. The structure is colored from red to blue from N-terminal tail to C-terminal tail. The predicted ligand binding site is shown in yellow, with the CG ligand presented inside. (b) Probabilities of natively entangled structure formation at the end of translation (*P*_Native_, blue) and ligand binding (*P*_Binding_, orange) vs. the interaction energy *ε*_*ij*_ (see Method). Error bars represent the 95% confidence intervals (CIs) estimated by bootstrapping. (c) Ligand binding probability vs. nascent chain length (top) and the averaged fraction of native contact formed in Domain III vs. nascent chain length with (red) and without (blue) ligand present (bottom). Transparent stripes represent 95% CIs estimated by bootstrapping. (f) Probabilities of forming misfolded entangled states P3 (orange), P4 (red), P8 (magenta), and native state P10 (blue) as a function of post-translational time for the slow DDLB variant with (right) and without (left) ligand present. Transparent stripes represent 95% CIs estimated by bootstrapping.

For DDLB, we selected the binding site on the second structure predicted by PEP-FOLD3 as the target site (Fig 5A), as the ligand bound to this structure can maximally occlude the formation of native contacts within the native closed loop. To evaluate its performance, we chose a binding strength of *ε*_*ij*_ = 1.0 kcal/mol for the protein folding simulations, as it is the lowest value in the affinity scan that yielded the highest number of correctly folded native entangled structures (Fig 5B).

During the co-translational simulations, the ligand quickly bound to the target and delayed the formation of native contacts within Domain III (Fig 5C). This resulted in a significant increase in the population of the native state (P10) at the end of the post-translational simulations, from 64% (95% CI [61%, 67%], without ligand) to 96% (95% CI [95%, 97%], with ligand), as shown in Fig 5D. The misfolded states P4 and P8 populations, which were observed in the absence of the ligand, neared zero in the presence of the ligand. Additionally, the misfolded state P3, which was unexpectedly enriched with the previous design method (Fig 3, panels f and g), was no longer observed.

**Fig 6:**
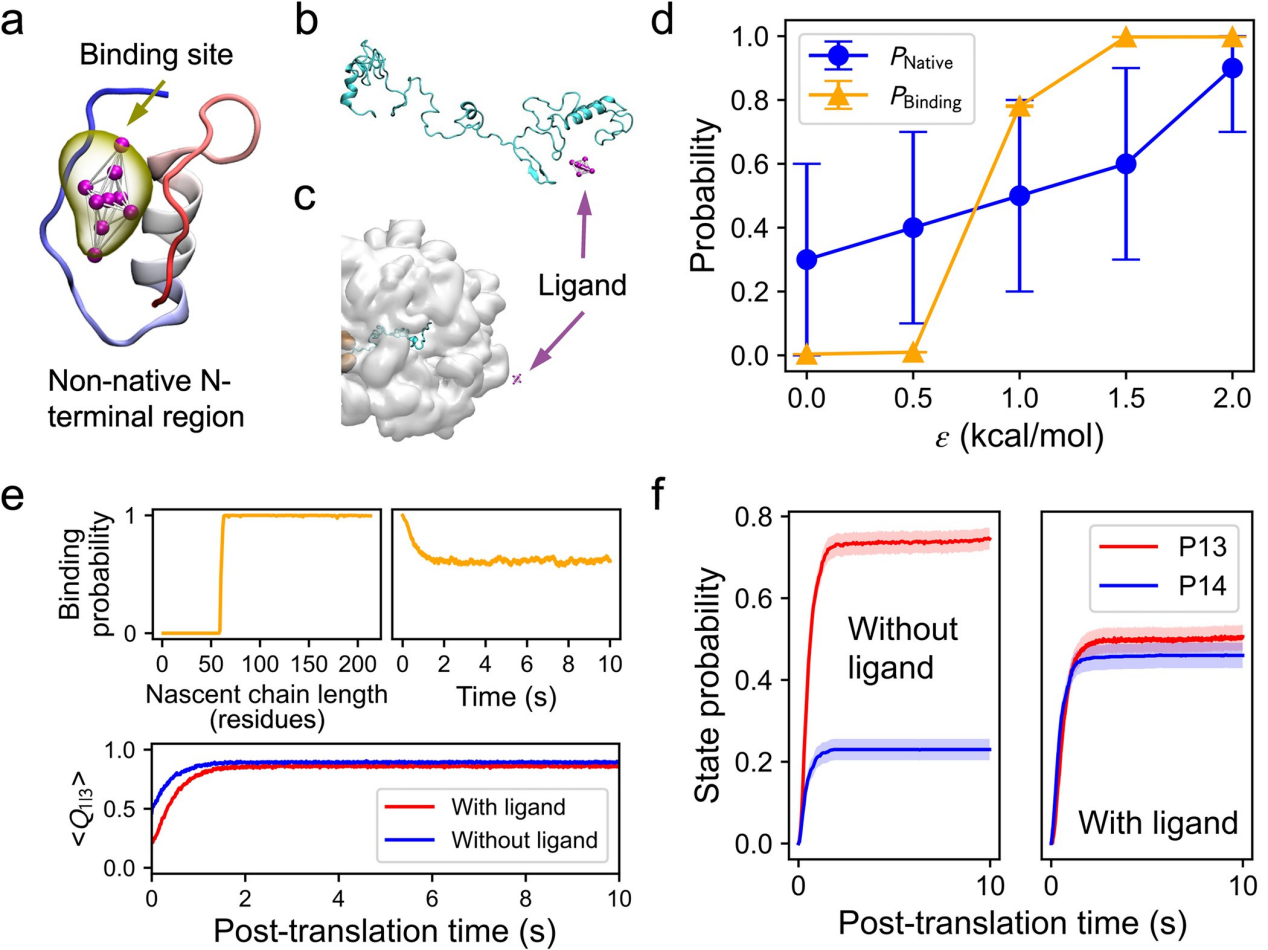
Ligand destabilizing native closed loop rescues misfolded CAT-III. (a) Predicted non-native structure of CAT-III N-terminal region. The structure is colored from red to blue from N-terminal tail to C-terminal tail. The predicted ligand binding site is shown in yellow, with the CG ligand presented inside. (b) Starting structure used in the binding affinity scan. (c) One of the starting structures of the RNC complex with a CG ligand present used in the co-translational folding simulations. (d) Probabilities of native state formation (*P*_Native_, blue) and ligand binding (*P*_Binding_, orange) vs. the interaction energy *ε*_*ij*_ (see Method). Error bars represent the 95% confidence intervals (CIs) estimated by bootstrapping. (e) Ligand binding probabilities vs. nascent chain length in the co-translational phase (left) and vs. time in the post-translational phase (right). Representative structures are presented on the top to depict the ligand binding. (e) Ligand binding probabilities vs. nascent chain length in the co-translational phase (top left) and vs. time in the post-translational phase (top right), and the averaged fraction of native contact formed between segments I and III (〈*Q*_1|3_〉) vs. post-translation time with (red) and without (blue) ligand present (bottom). Transparent stripes represent 95% CIs estimated by bootstrapping. (f) Probabilities of forming the misfolded entangled state P13 (red) and native state P14 (blue) vs. post-translational time for the fast CAT-III variant with (right) and without (left) ligand present. Transparent stripes represent 95% CIs estimated by bootstrapping.

For CAT-III, we identified the binding site connecting both tails of the segment as the best candidate to effectively sequester this native loop region. Therefore, we selected the 5^th^ non-native structure predicted by PEP-FOLD3 (Fig 6A). We performed an affinity scan starting from a full-length, unfolded CAT-III structure off the ribosome (Fig 6B). We chose a binding strength of *ε*_*ij*_ = 1.0 kcal/mol for subsequent simulations, which allowed for multiple binding/unbinding events and resulted in at least half of the folding trajectories reaching the native state (Fig 6D). The protein folding simulations of the fast CAT-III variant were started in the co-translational phase (100 trajectories) when 60 residues had been translated and the target N-terminal region had emerged on the ribosome (Fig 6C). The ligand bound to the nascent chain quickly and remained bound with a probability of over 0.99 until the end of translation (Fig 6E). During post-translational folding, the binding probability decreased to about 0.60 at the end (Fig 6E) due to the spontaneous folding of the N-terminus. Early binding of the ligand to the N-terminal region significantly delayed the native loop closing, as demonstrated by the lower 〈*Q*_1|3_〉 values for the protein with ligand present Fig 6E. We observed a significant increase in the probability of the predominate native folding pathway involving passage through state P3, from 0.17 (95% CI [0.15, 0.19]) to 0.41 (95% CI [0.38, 0.44]) upon ligand binding. This increased flux to state P3 also indicates that significantly more proteins were able to delay the closing of the native loop with the assistance of ligand binding. The delayed loop closing reduced the probability of forming the near-native misfolded state P13 and increased the probability of forming the native state P14 by about two-fold compared to the system without the ligand, from 23% (95% CI [20%, 26%]) to 46% (95% CI [43%, 49%]) (Fig 6F).

These results are consistent with our hypothesis. Ligand binding to non-native tertiary structure in both DDLB and CAT-III early in the folding process destabilized the native closed loop, thereby delaying loop closure, and affording more time for the threading segment to be positioned for the loop to wrap around it and achieve its native structure.

### FDA-approved drugs that might avoid misfolding

Instead of designing new drug candidates we asked whether we could identify existing drugs that could potentially avoid this type of misfolding. We conducted a virtual screening of 2,056 Federal Drug Administration (FDA)-approved drugs. We looked for drugs that could bind to the predicted non-native structures while simultaneously having less affinity for the native, folded proteins thereby increasing specificity. We ranked the FDA-approved drugs by the difference between their docking score to the non-native structure versus to the native structure and selected the top 5 drugs that showed the largest difference. These top 5 drugs for DDLB and CAT-III are reported in Tables 1 and 2, respectively.

**Table 1:** Top 5 drug candidates for DDLB that showed the strongest binding affinity to the non-native structure and weaker affinity to the native structure.

ID	Compound name	Docking score[15] to non-native structure	Docking score to native structure
1.	Digoxin	-82.18	-71.56
2	Aprepitant	-93.29	-84.59
3	Delavirdine	-73.09	-66.1
4	Sirolimus	-73.64	-66.78
5	Tyloxapol	-67.79	-61.77

**Table 2:** Top 5 drugs for CAT-III that showed the strongest binding affinity to the non-native structure and no binding affinity to the native structure.

ID	Compound name	Docking score[15] to non-native structure	Docking score to native structure
1.	Latanoprost acid	-100.93	0
2.	Ezetimibe	-94.91	0
3.	Canagliflozin	-94.65	0
4.	Epoprostenol	-94.61	0
5.	Valsartan	-94.51	0

We conducted blind docking simulations to investigate the potential binding sites of the 5 candidate drugs on early stage folding intermediate structures. For CAT-III, we randomly sampled 10 intermediate structures from the first two metastable states (as shown in S2 Fig) clustered using the first 50 ns data in the post-translational folding trajectories in the presence of the CG ligand. While for DDLB, we used the co-translational trajectories at nascent chain lengths 195 to 210 in the presence of the CG ligand.

For each combination of the intermediate structure and the candidate drug, we obtained the top 5 binding poses from blind docking (as shown in S3 Fig for DDLB and S4 Fig for CAT-III). We selected the on-target binding pose with the best binding score across all candidates for each intermediate structure (as described in the Methods section *Blind docking*). The on-target binding poses occurred in all 10 intermediate structures for DDLB and 8 of the 10 structures for CAT-III, which suggests these top 5 drugs have the potential to robustly bind to the ensemble of non-native tertiary structures.

We also tested whether the candidates could bind to the target segments in the native protein structures. As shown in S5 Fig, none of the candidates had binding poses that bound to the corresponding segments in the natively folded CAT-III and DDLB, respectively, in the top 5 predictions generated by the blind docking simulations. This result is consistent with the prediction obtained from the virtual screening that these drugs show high specificity for the target binding structure.

Next, we evaluated the residence time of the on-target binding poses by performing all-atom MD simulations for the on-target protein-ligand complex structures with the best binding score (see Methods section *All-atom simulations for protein-ligand complex*). We found 50% and 62.5% of the DDLB and CAT-III trajectories, respectively, still had the ligand bound on-target in the last half of 1-microsecond simulations, indicating these might be stable binding poses for these candidates (see S6 Fig).

These results suggest it might be possible to leverage existing FDA approved drugs to correct protein misfolding involving changes of native entanglements and restore protein function.

## Discussion

We have explored two alternative drug design strategies to avoid misfolding involving non-covalent lasso entanglements *in silico* that could be useful in treating loss-of-function diseases where the amount of functional protein is below a threshold concentration. No drug-design strategies currently exist as such misfolding has only recently been suggested to occur [1–3]. In proteins CAT-III and DDLB we observed in our model that the misfolding mechanism involved the failure-to-form a native entanglement and the concomitant formation of a non-native non-covalent lasso. The predominant pathway for properly folding the native entanglement is for the loop to wrap and close around the threading segment (also known as the “embracement” mechanism [16]), as opposed to the loop closing first and then the threading segment through the loop. The latter pathway, involving direct loop piercing (plugging) and/or slipknotting [16,17], was observed only in CAT-III. This distinction arises from CAT-III’s distinctive structural attributes—a large native loop comprising 74 residues and a relatively shallow threading segment positioned 23 residues away from the C-terminus. In contrast, DDLB, characterized by a shorter native loop of 49 residues and a threading segment situated deep in the primary structure 151 residues from the C-terminus, precludes the possibility of either direct piercing or slipknotting during both co- and post-translational folding processes.

Folding pathway analysis led us to create and test two strategies: stabilizing the native entanglement during folding by creating *in silico* compounds that simultaneously bind segments of the loop and thread; and slowing down (destabilizing) loop closure by a compound that stabilized non-native tertiary structure involving the loop and thereby allowing more time for the threading segment to be properly positioned before loop closure. The first design strategy failed to increase the folding yield for the two proteins. While the second strategy succeeded.

In small-molecule drug design it is common to target a protein’s native functional state for drug binding [18]. Binding in this way thermodynamically stabilizes the native state and therefore can help restore function in proteins that tend to misfold. We therefore found it surprising that designing *in silico* compounds that targeted the native entangled structure did not increase proper folding. At the molecular level, this failure arose because the drug promoted folding of the loop before the threading segment was properly position, meaning to reach the native state the much slower pathway must be taken of the thread piercing an already closed loop. We suspect this design approach will fail for many different proteins because the native topology of around half of all globular proteins in *E*. *coli*, yeast, and human proteomes contain such entanglements.

Our second design strategy is less common in the drug development field: we targeted the binding of transient, non-native tertiary structure. To our knowledge, there are no FDA-approved drugs that are designed to target a transient, non-native tertiary structure. At a practical level, this is more difficult to do as such structures are fleeting and not easy to structurally characterize making it difficult to identify binding surfaces to design drugs to complement. In a computer it is, of course, much easier to do these things. And when we did this, it worked very well as demonstrated by increasing the native state population two-fold. This represents one of the likely challenges that will be faced in taking this design strategy into the lab. When attempting this approach in the wet lab, lessons might be drawn from research communities that have been attempting to design small molecules to bind biological condensates–structurally ill-defined amalgamations of RNA and protein in cells undergoing liquid-liquid phase separation–for the purpose of modulating their formation [19–21] and those to bind protein folding intermediates [22,23]. That communities’ years of efforts to target the extensive transient tertiary structure present in these mixtures might be transferable to this new class of misfolding.

The last portion of our study offers a partial solution to this challenge. Specifically, through virtual screening and docking of FDA-approved drugs to the misfolded structures seen in our simulations, we identified 5 drug candidates for both proteins that computationally are predicted to have some of the highest specificity for the non-native tertiary structure. This suggests the possibility that designing potent small molecules may be achievable. Testing these candidates in the wet lab on DDLB and CAT-III will also be important in future studies.

Targeted protein degradation is an active area of therapeutic development for gain-of-function diseases in which biologics are designed for the purpose of eliminating disease-associated proteins via enhanced degradation [24]. While this approach often involves proteolysis-targeting chimeras [25] that simultaneously bind a target protein and E3 ubiquitin ligase [26] that promotes degradation via the ubiquitin pathway, we speculate our first design strategy also has the potential to contribute to promote targeted protein degradation. Specifically, we saw that in our attempts to stabilize the native entanglement in the first design strategy we promoted misfolding into specific misfolded states. Misfolded structures are more likely to be degraded than properly folded structures. Therefore, for proteins that contribute to gain-of-function diseases and also have an entanglement in their native state, it might be the case that designing compounds that aim to bind native entanglements during folding may promote misfolding and degradation.

There is a body of computational and experimental evidence consistent with the existence of this type of misfolding, making it worthwhile to explore the possibility of drug interventions. In our previous modeling of 122 proteins from *E*. *coli* using the same coarse-grained force field, we observed that half of them had subpopulations of misfolded states characterized by changes in non-covalent entanglement status [1]. Moreover, our analysis of DE Shaw’s unrestrained, all-atom protein folding simulations in explicit solvent, encompassing various proteins (albeit small), demonstrated the transient occurrence of this type of misfolding [27]. In the case of CAT-III and DDLB, our all-atom simulations in explicit solvent indicated that the misfolded states served as long-lived kinetic traps, estimated to persist for at least two hours [3]. Complementary to our computational findings, predictions from this model concerning CAT-III and DDLB were validated through activity measurements as well as Limited-Proteolysis Mass Spectrometry data, indicating these entangled misfolded states are real [1,3,27].

The coarse-grained model we use is at a resolution of 3.8 Å and treats residues as varying size spheres. If the formation of non-covalent lasso entanglements depends sensitively on such details, our simulation model could over or underestimate the prevalence of this type of misfolding. We anticipate that this sensitivity will be a function of the loop size of the lasso. The formation of entanglements involving short loops, which will have smaller free volumes to accommodate the threading segment, are likely to be more sensitive to shape and excluded volume details of the model. Larger loops less so. Therefore, the results of this study should be viewed as identifying a molecular scenario that is possible (*i*.*e*., avoiding such misfolding with small molecules), and little weight should be attached to exact population numbers, *etc*.

The next steps in this line of research are clear. Identifying proteins associated with loss-of-function or gain-of-function diseases that might be caused by failure-to-form mechanisms is a high priority as this would provide a candidate list of drug targets. Followed by high-throughput experimental characterization to narrow this list down to those protein candidates that are likely to misfold via a change of entanglement. And finally, the design and experimental testing of compounds to promote folding for loss of function diseases or promote misfolding and presumably degradation, for gain of function diseases. We believe this line of inquiry could open many new avenues for therapeutic treatment for a potentially wide range of diseases.

## Methods

### Folding/misfolding pathways analysis

We analyzed the post-translational folding trajectories of CAT-III and DDLB obtained from our previous work [3]. The pathways were identified using the same algorithm [3] for all trajectories of the fast and slow variants (2,000 trajectories in total). In brief, we first obtained the discrete trajectories by assigning the metastable states on each of the structures in the trajectories. For each discrete trajectory, we then constructed pathway that has no loop on the route and only records the on-pathway states for each discrete trajectory (details can be found in Ref. [3]).

To facilitate the pathway analysis, we calculated the fraction of native contacts (*Q*), fraction of gain of non-native entanglements (*G*_gain_) and fraction of loss of native entanglements (*G*_loss_), as per the following equations [3]:

$$Q_{I|J}=\frac{\sum_{i\in I}\sum_{j\in J}\Theta (i,j|\mathrm{Current})}{\sum_{i\in I}\sum_{j\in J}\Theta (i,j|\mathrm{Native})},$$ (Eq 1)

$$G_{gain}=\frac{1}{N}\sum_{\left(i,j\right)}\Theta (\left(i,j)\in \mathrm{nc}\cap |g(i,j)|>|g^{\mathrm{native}}(i,j)\right|),$$ (Eq 2)

and

$$G_{loss}=\frac{1}{N}\sum_{\left(i,j\right)}\Theta (\left(i,j)\in \mathrm{nc}\cap |g(i,j)|<|g^{\mathrm{native}}(i,j)\right|).$$ (Eq 3)

In Eq 1, *I* and *J* are two sets of residues, with *i* and *j* are the residue indices satisfying *j*>*i*+3; *Θ*(*i*, *j*|Current) and *Θ*(*i*, *j*|Native) are step functions that equal 1 when residue *i* and *j* have native contact and 0 when *i* and *j* do not have native contact in the current structure and native structure, respectively. Native contacts are considered formed when the distance between the Cα atoms of residues *i* and *j* does not exceed 1.2 times their native distance and the native distance does not exceed 8 Å. In Eqs 2 and 3, (*i*, *j*) is one of the native contacts in the native crystal structure; *nc* is the set of native contacts formed in the current structure; *g*(*i*, *j*) and *g*^native^(*i*, *j*) are, respectively, the total linking number of the native contact (*i*, *j*) in the current and native structures estimated using the Supplementary Eq 16 in Ref. [3]; *N* is the total number of native contacts within the native structure; and the selection function *Θ* equals 1 when the condition is true and 0 when it is false.

### Generic coarse-grained model of small molecule ligands

To develop a generic coarse-grained (CG) model for small molecule ligands, we first examined the size distribution of FDA-approved small molecule drugs. We obtained 3D structures of 2,056 molecules from the e-Drug3D database [28] and computed their principal axes. Then, we projected the Cartesian coordinates of each molecule onto its principal axes and determined its dimension on each principal axis as the maximum distance between two atoms along that axis. We used the median dimensions across all FDA-approved small molecule drugs along the three principal axes (i.e., 11.46 Å × 6.11 Å × 3.35 Å) to create the geometry of the generic CG model.

We created a model with an octahedral geometry consisting of 9 interaction sites (CG beads), with one site located at the origin and the other eight distributed along the x, y, and z axes. The longest axis contains four interaction sites, while each of the other two axes contains two sites. The interaction sites on each axis evenly divide the corresponding dimension. The energy term for both intra- and inter-molecular interactions within the ligands is described as

$$E_{\mathrm{tot}}^{L-L}=\sum_{i\in \{L\}}K_{b}{\left(b_{i}-b_{0}\right)}^{2}+\sum_{\begin{array}{c} i,j\in \{L\},\\ i\neq j \end{array}}\varepsilon_{ij}[13{\left(\frac{R_{ij}}{r_{ij}}\right)}^{12}-18{\left(\frac{R_{ij}}{r_{ij}}\right)}^{10}+4{\left(\frac{R_{ij}}{r_{ij}}\right)}^{6}],$$ (Eq 4)

where, *b*_0_ is the bond length between two interaction sites within a ligand molecule; *K*_b_ is the force constant of 50 kcal/mol/Å^2^. We incorporated a weak repulsive force between any two interaction sites, which is similar to the ’non-native’ interaction forces in our previous model [3], where $\varepsilon_{ij}=\sqrt{\varepsilon_{i}∙\varepsilon_{j}}$ and *R*_*ij*_ = *R*_*i*_+*R*_*j*_. Here, *ε*_*i*_ was set to 0.000132 kcal/mol and *R*_*i*_ was set as the median value of the amino acid residue parameters, which is 3.415358 Å. As these interaction sites have zero charge, there are no electrostatic interactions among them. The molecular weight of the ligand was set as the average molecular weight of the FDA approved drugs (388.46 Daltons). We assigned the same mass to all interaction sites, evenly dividing the molecular weight of the ligand.

### Coarse-grained simulation for protein folding with presence of ligand

We modified the previously developed CG model [3] to simulate protein co- and post-translational folding in the presence of ligands. To do this, we introduced energy terms for the nonbonding interactions between the ligand and the protein (or nascent chain, denoted as $E_{\mathrm{tot}}^{\mathrm{NC}‐L}$) or the ribosome ($E_{\mathrm{tot}}^{R‐L}$). These energy terms can be described as follows:

$$\left\{ \begin{array}{c} E_{\mathrm{tot}}^{\mathrm{NC}‐L}=\sum_{\begin{array}{c} i\in \{\mathrm{NC}\},\\ j\in \{L\} \end{array}}\varepsilon_{ij}[{\left(\frac{R_{ij}}{r_{ij}}\right)}^{12}-2{\left(\frac{R_{ij}}{r_{ij}}\right)}^{6}],\\ E_{\mathrm{tot}}^{R-L}=\sum_{\begin{array}{c} i\in \{R\},\\ j\in \{L\} \end{array}}\varepsilon_{ij}[13{\left(\frac{R_{ij}}{r_{ij}}\right)}^{12}-18{\left(\frac{R_{ij}}{r_{ij}}\right)}^{10}+4{\left(\frac{R_{ij}}{r_{ij}}\right)}^{6}]. \end{array}\right.$$ (Eq 5)

For interactions between the ligand and the binding site residues, we adjusted *ε*_*ij*_ to produce a reasonable binding affinity (see Method section *Binding affinity scan*), while *R*_*ij*_ was set to reproduce a predefined binding pose (see Method section *Binding site prediction*). For the interactions between the ligand and other protein residues, as well as those between the ligand and ribosome, we computed *ε*_*ij*_ and *R*_*ij*_ in the same manner as those in $E_{\mathrm{tot}}^{L‐L}$. No distance cutoff or switching function was applied to $E_{\mathrm{tot}}^{\mathrm{NC}‐L}$, whereas the same distance cutoff and switching function were applied to $E_{\mathrm{tot}}^{L‐L}$ and $E_{\mathrm{tot}}^{R‐L}$ as those used in the previous model [3]. All the other force field parameters in this model were taken from the previous parameter set [3].

To improve the possibility of ligand binding, we restrained the ligand within a spherical boundary around the protein by applying the following potential:

$$E_{\mathrm{sp}}=\left\{ \begin{array}{l} K_{\mathrm{sp}}{\left(d-d_{0}\right)}^{2},if\;d>d_{0};\\ 0,if\;d\leq d_{0}; \end{array}\right.$$ (Eq 6)

where *K*_sp_ is the force constant and *d*_0_ is the radius of the spherical boundary. For the co-translational simulations, *d*_0_ is set to 100 Å and *d* is set as the distance between an interaction site of the ligand and the spherical center, which is placed at the coordinate (160, 0, 0) Å in the system. For post-translational simulations, *d*_0_ is set to 200 Å and *d* is set as the distance between the center of mass (COM) of the ligand and the COM of the protein. *K*_sp_ was set to 0.1 kcal/mol/Å^2^ for both simulation phases.

Simulations for co- and post-translational folding in the presence of ligand were performed via Langevin dynamics with a collision frequency of 0.05 ps^−1^ and a time step of 15 fs using OpenMM [29]. For co-translational folding, simulations were initiated from the nascent chain length of 60 and 190 residues for CAT-III and DDLB, respectively, at which point the target segments began to emerge on the ribosome, using ribosome-nascent chain (RNC) complex structures obtained from previous simulations for the fast variant of CAT-III and slow variant of DDLB [3]. We performed 100 independent simulations for co-translational folding and 1,000 simulations for post-translational folding (10 replicate simulations starting from each co-translational trajectory) for 10 seconds on the experimental timescale (approximately 2 microseconds on the simulation timescale). A CG ligand was placed near the exit of the ribosomal exit tunnel at a random position. More information on the timescale mapping and RNC complex model setup can be found in the previous study [3].

### Binding affinity scan

To ensure a reasonable binding affinity for the protein-ligand complex, we conducted a binding affinity scan by setting up a series of CG simulations with different *ε*_*ij*_ values for the interactions between the ligand and the binding site residues, specifically, *ε*_*ij*_ = 0, 0.5, 1.0, 1.5 and 2.0 kcal/mol. For each simulation system, we performed 10 independent simulations, each running for 1 microsecond. To assess the binding affinity of each system, we computed the probability of trajectories that formed the native entanglements at the end of the simulation (*P*_Native_) and the probability of ligand binding (*P*_Binding_). Formation of native entanglements was identified by observing a *G* value (fraction of native contacts with non-native entanglements [3]) below 0.02 for a full-length CAT-III structure and a *G*_gain_ value below 0.002 for a partially synthesized DDLB structure, averaged over the last 100 frames. The binding event was identified as the case that the shortest distance between the ligand and protein was no greater than 8 Å. The final *ε*_*ij*_ value was taken as the smallest one that yielded *P*_Native_≥0.5 (good rescuing performance) and 0.5<*P*_Binding_<1.0, (moderate binding affinity with multiple binding and unbinding events observed) for CAT-III and the smallest one that yielded *P*_Native_ = 1 for DDLB.

The initial complex structure was created by randomly placing a single ligand CG molecule near the binding site. The initial protein and RNC conformations were obtained from our previous simulations for the fast CAT-III variant and slow DDLB variant, respectively [3].

### Protein segment structure prediction

To predict the possible structures formed during the folding of the DDLB and CAT-III segments, we used three well-known sequence-based protein/peptide structure prediction tools, AlphaFold2[10,11], PEP-FOLD3[12] and QUARK [13,14]. The peptide sequence of the *E*. *coli* DDLB residues 126 to 174 (LSDKQLAEISALGLPVIVKPSREGSSVGMSKVVAENALQDALRLAFQHD) and the N-terminal 35 residues of the *E*. *coli* CAT-III (MNYTKFDVKNWVRREHFEFYRHRLPCGFSLTSKID) were used as the input sequences for the structure prediction with default settings. In each sequence, the top 5 predicted structures from each tool were compared to determine the set of structures for binding site identification.

### Binding site prediction

AutoSite [9] was used to identify possible binding sites on a given structure with default settings. The most reasonable binding site was chosen to build the structural-based CG model for the protein-ligand complex. The initial complex structure was created by placing the generic CG ligand on the binding site with an arbitrary pose, where the ligand had a moderate distance from the binding site residues.

### Virtual screening of FDA-approved drugs

To identify potential drugs that can rescue the misfolded entangled protein, we conducted a virtual screening of 2,056 FDA-approved drugs using the e-LEA3D webserver [30]. We used the predicted non-native structure of the segments in CAT-III and DDLB and the entire native structures as the target macromolecule for the virtual screening, respectively. For the both non-native and native structures, the binding site position was established as the one predicted by AutoSite [9]. A binding site radius of 15 Å was used in both virtual screening calculations. For each protein, the top 5 drugs that had the largest difference (more negative) between the PLANTS docking score [15] to the non-native segments and that to the native structure were selected to further evaluation.

### Blind docking

Blind docking simulations were performed to evaluate the binding of 5 candidate drugs on early-stage folding intermediate states for DDLB and CAT-III. The simulations were conducted using the CB-Dock2 webserver [31]. For each protein, 10 representative structures were used as targets, obtained from the first two metastable states (five representative structures drawn from each) clustered using the first 50 ns trajectories of post-translational simulations of CAT-III and the co-translational trajectories from length 195 to 210 of DDLB, respectively, with the generic ligand bound and back-mapped from the CG model to the atomic resolution [3]. For DDLB structures, the last 20 amino acid residues were removed as they are fully buried in the ribosome exit tunnel. For each combination of the drug and the target protein structure, the top 5 binding poses were generated. We selected the best candidate drug and its on-target binding pose with the lowest binding energy from each target intermediate structure. The on-target binding pose was determined as the one in which no less than 80% of the contacts between the ligand and the protein are in the target region. In addition, for CAT-III we requested at least both ends of the segment (residues 1 to 12 and residues 25 to 35) make contacts with the ligand, while for DDLB, we requested at least the central beta-strand (residues138 to 147) makes contacts with the ligand. Contacts were considered formed when the shortest distance between the ligand and the protein residue is less than 4 Å. This complex structure was then used as the initial protein-ligand complex for all-atom MD simulations.

### All-atom simulations for protein-ligand complex

For each system, the complex was embedded in a periodic TIP3P water box. Several counter ions were added to neutralize the system. The Particle Mesh Ewald (PME) method [32] was used to calculate the long-range electrostatic interactions with a 10 Å cutoff. The solvated system was relaxed through a 5000-step energy minimization, an NVT ensemble simulation stepwise heating the temperature to 310 K for 20 ps, an NPT ensemble simulation relaxing the water box for 600 ps, and an NVT ensemble production simulation for 1 microsecond. Except for the production simulations, the protein Cα atoms and the ligand heavy atoms were restrained at the starting position using a force constant of 100 kcal/mol/Å^2^. The NPT ensemble simulations were performed at 310 K temperature and 1 bar pressure via Langevin dynamics (the collision frequency is 1.0 ps-1), with a coupling constant of 0.2 ps for both parameters. The lengths of the bonds involving hydrogen were constrained, which ensures the integral timestep to be 2 fs. The production simulations were performed by OpenMM [29] on GPUs, while the other steps were performed by Amber17[33]. The simulation systems were parameterized by the ff14SB protein force field [34]. The ligands were parameterized by the general force field gaff [35]. The atomic charges of the ligands were estimated as the AM1-BCC charges [36,37] using Amber Tools17[33]. As the DDLB structures are all nascent chain proteins, the Cα atom of the last residue was harmonically restrained at the initial position with a force constant of 1 kcal/mol/Å^2^. For a single frame, the ligands were considered bound on-target only if more than half of the contacts are on the target segment. Contacts were considered formed when the shortest distance between the ligand and the protein residue is less than 4 Å.

## Supporting information

S1 Fig Segment structure prediction and binding site identification for DDLB and CAT-III. (a) Pairwise RMSD between the native DDLB segment (residues 126 to 174, obtained from PDB 4C5C) structure and those predicted by AlphaFold2, PEP-FOLD3, and QUARK. (b) Superimposed structures obtained from each prediction for DDLB, colored from red to blue from N-terminal tail to C-terminal tail. (c) Non-native structures of DDLB segment with successfully identified ligand binding sites (shown in yellow). The binding site on PEP-FOLD3 #2 was selected for DDLB drug design. (d) Pairwise RMSD between the native CAT-III segment (residues 1 to 35, obtained from PDB 3CLA) structure and those predicted by AlphaFold2, PEP-FOLD3, and QUARK. (e) Superimposed structures obtained from each prediction for CAT-III, colored from red to blue from N-terminal tail to C-terminal tail. (f) Non-native structures of CAT-III segment with successfully identified ligand binding sites (shown in yellow). The binding site on PEP-FOLD3 #5 was selected for CAT-III drug design.
(TIF)

S2 Fig Clustering of metastable states using the first 50 ns of post-translational folding trajectories of CAT-III with ligand bound. (Left) The -ln[P] surface plotted over parameter *G* and *Q*_act_. (Right) Metastable state distributions on the -ln[P] surface. A total of 10 metastable states were clustered, with the first two states (S1 and S2) being the most predominant.
(TIF)

S3 Fig Blind docking results for representative DDLB structures. The top 5 binding poses are presented for each nascent chain protein structure and each candidate drug. The binding score (Autodock vina score) is shown near each binding pose. The scores for the binding poses that are located on target are colored in red. The protein structures are colored from red to blue from N-terminal tail to C-terminal tail. The C-terminal 20 amino acids in the nascent chains that are embedded in the ribosome exit tunnel were removed in the blind docking.
(TIF)

S4 Fig Blind docking results for representative CAT-III structures. The top 5 binding poses are presented for each protein structure and each candidate drug. The binding score (Autodock vina score) is shown near each binding pose. The scores for the binding poses that are located on target are colored in red. The protein structures are colored from red to blue from N-terminal tail to C-terminal tail. Protein structures #3 and #9 have no on-target binding pose detected.
(TIF)

S5 Fig Blind docking results for candidate drugs on the native structures of DDLB and CAT-III. The protein structures are obtained from the PDBs 4C5C (chain B) and 3CLA (chain A), respectively, colored from red to blue from N-terminal tail to C-terminal tail. The top 5 binding poses for each of the 5 candidates are presented. No binding pose was found at the target segments.
(TIF)

S6 Fig **Fraction of on-target contacts formed between the ligand and protein structures ((a) DDLB, (b) CAT-III) in the all-atom simulations.** 50% of the DDLB trajectories and 62.5% of the CAT-III trajectories have average fraction of on-target contacts greater than 0.5 within the last 500 ns (Complex #1, #5, #6, #8 and #10 of DDLB; Complex #1, #4, #5, #6 and #10 of CAT-III).
(TIF)
