# Supplementary figures and images for: It is theoretically possible to avoid misfolding into non-covalent lasso entanglements using small molecule drugs
Source: PLoS Comput Biol. 2024 Mar 12;20(3):e1011901. doi: 10.1371/journal.pcbi.1011901 (PMC10931463; doi:10.1371/journal.pcbi.1011901)

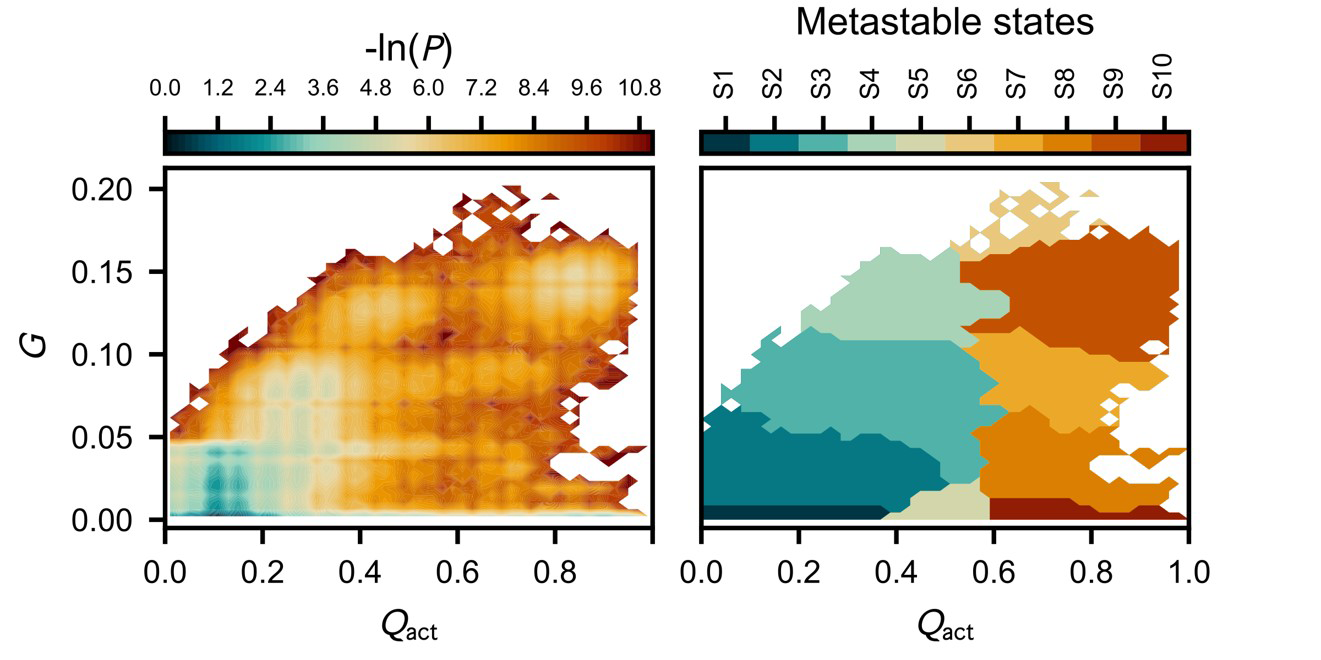

Supplement: S2 Fig — (Left) The -ln[P] surface plotted over parameter G and Qact. (Right) Metastable state distributions on the -ln[P] surface. A total of 10 metastable states were clustered, with the first two states (S1 and S2) being the most predominant. (TIF) [file pcbi.1011901.s002.tif]

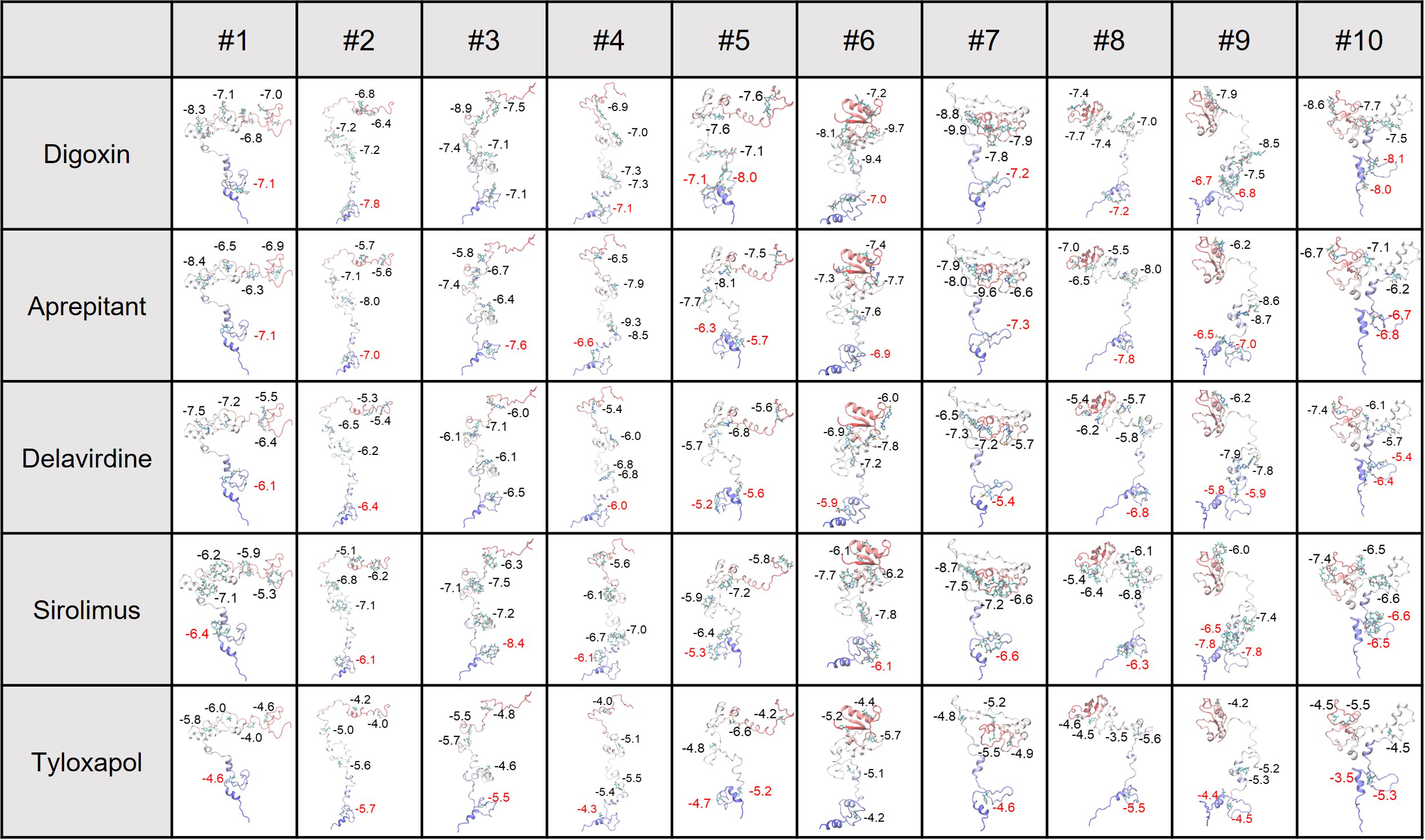

Supplement: S3 Fig — The top 5 binding poses are presented for each nascent chain protein structure and each candidate drug. The binding score (Autodock vina score) is shown near each binding pose. The scores for the binding poses that are located on target are colored in red. The protein structures are colored from red to blue from N-terminal tail to C-terminal tail. The C-terminal 20 amino acids in the nascent chains that are embedded in the ribosome exit tunnel were removed in the blind docking. (TIF) [file pcbi.1011901.s003.tif]

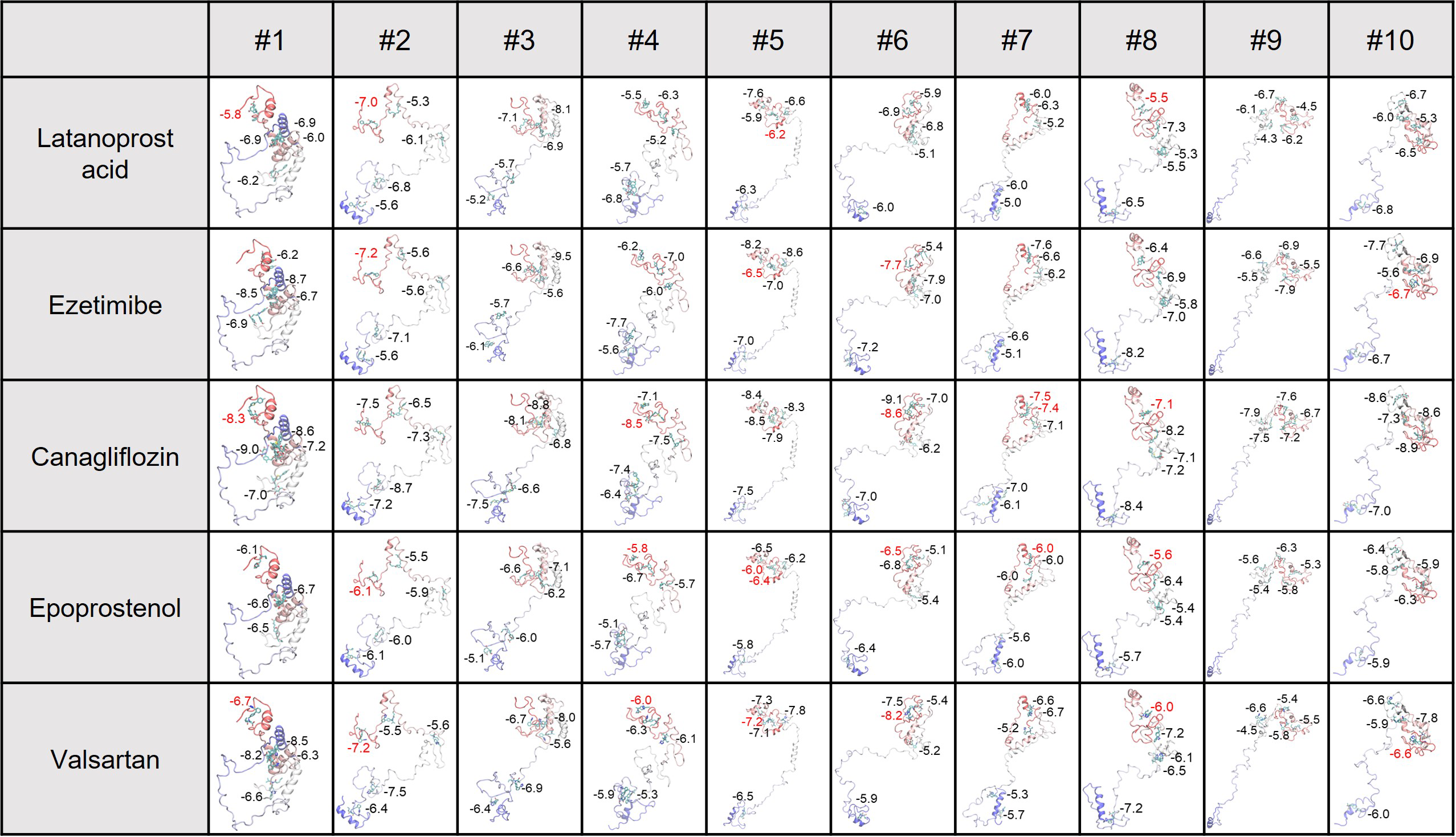

Supplement: S4 Fig — The top 5 binding poses are presented for each protein structure and each candidate drug. The binding score (Autodock vina score) is shown near each binding pose. The scores for the binding poses that are located on target are colored in red. The protein structures are colored from red to blue from N-terminal tail to C-terminal tail. Protein structures #3 and #9 have no on-target binding pose detected. (TIF) [file pcbi.1011901.s004.tif]

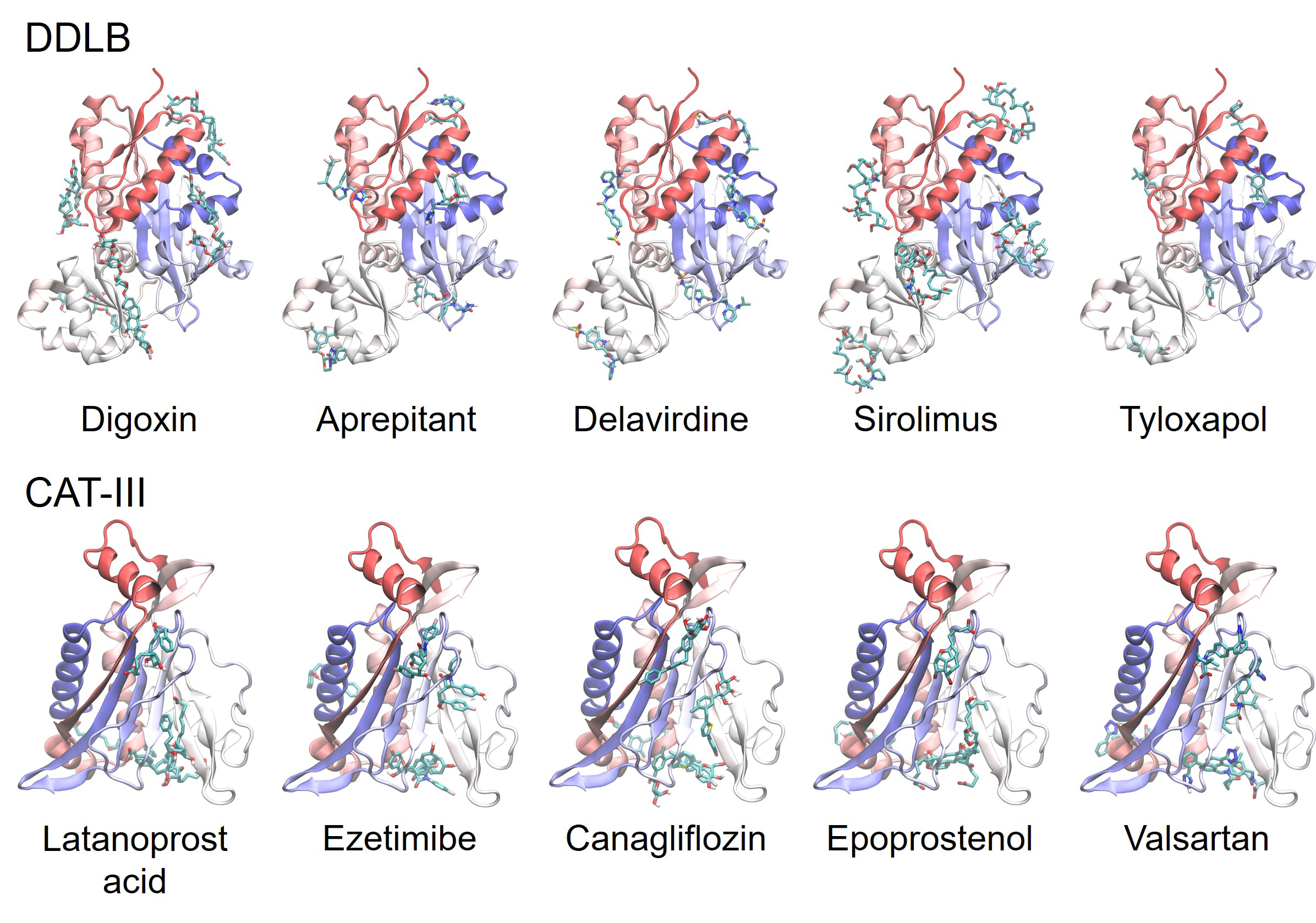

Supplement: S5 Fig — The protein structures are obtained from the PDBs 4C5C (chain B) and 3CLA (chain A), respectively, colored from red to blue from N-terminal tail to C-terminal tail. The top 5 binding poses for each of the 5 candidates are presented. No binding pose was found at the target segments. (TIF) [file pcbi.1011901.s005.tif]

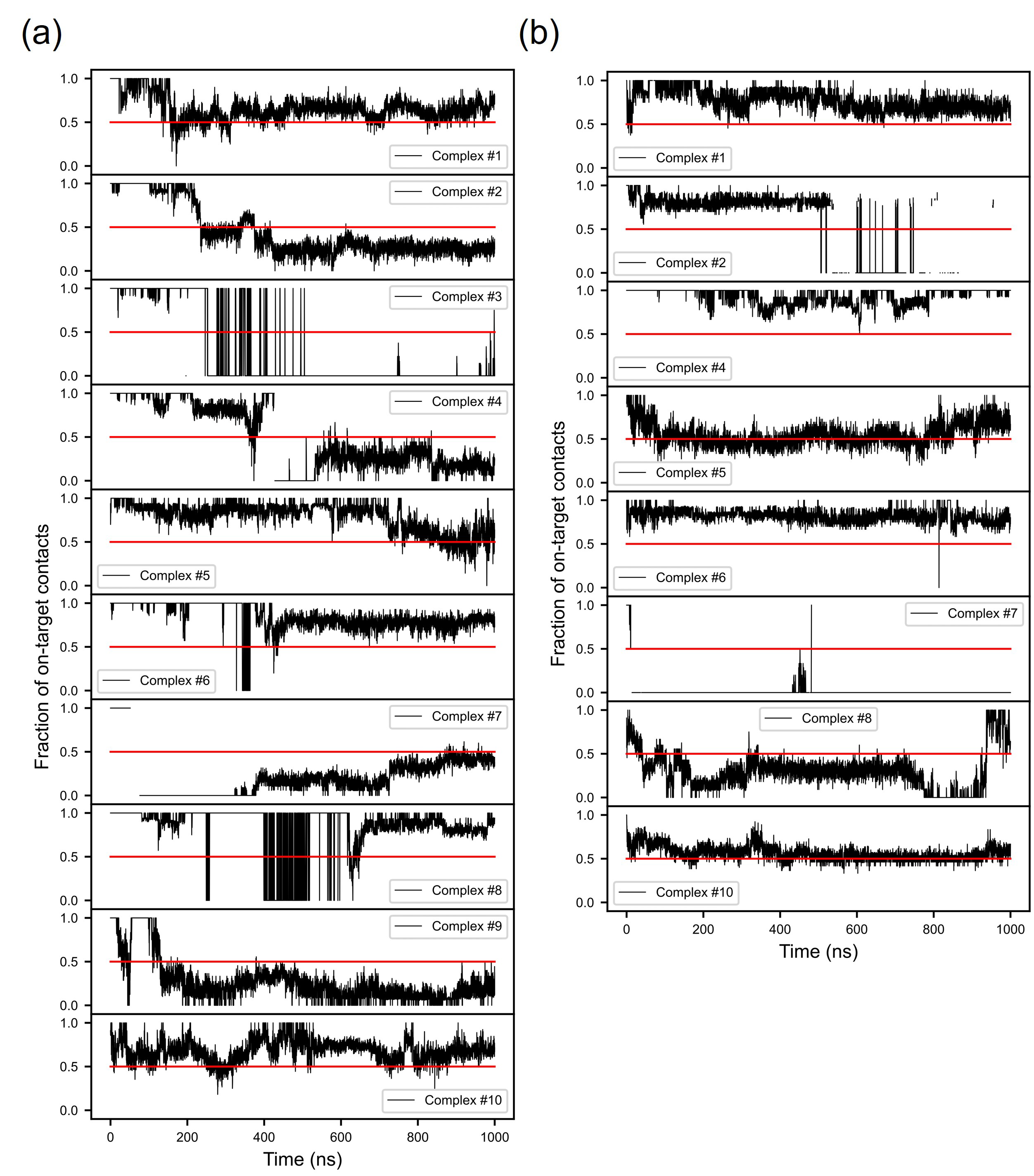

Supplement: S6 Fig — Fraction of on-target contacts formed between the ligand and protein structures ((a) DDLB, (b) CAT-III) in the all-atom simulations. 50% of the DDLB trajectories and 62.5% of the CAT-III trajectories have average fraction of on-target contacts greater than 0.5 within the last 500 ns (Complex #1, #5, #6, #8 and #10 of DDLB; Complex #1, #4, #5, #6 and #10 of CAT-III). (TIF) [file pcbi.1011901.s006.tif]
